# Supplementary material for: The Tower Building Task: A Behavioral Tool to Evaluate Recreational Risk-Taking
Source: Behav Sci (Basel). 2022 Sep 8;12(9):325. doi: 10.3390/bs12090325 (PMC9495860; doi:10.3390/bs12090325)

Supplementary Material

S1. Instructions for each TBT condition……………………………………………………………………………2-3

S2. Criteria for considering the involuntary loss of height as a collapse…………………………………………..….3

S3. How the record was determined? ...…………………………………………………………………………………3

S4. Translation in Spanish of the Sensation Seeking Scale...………………………………………………………4-6

S5. Additional descriptors of behavior in the TBT……………………………………………………………….….7

S6. Scatter plots of the non-significant associations between fixed height gain and the two other risk-related measures……………………………………………………………………………………………………....8-10

S1. Instructions in Spanish of the Tower Building Task in each of the four conditions

I) Baseline

(i) El objetivo es construir la torre más alta que puedas en un periodo de 10 minutos.
(ii) Utiliza los bloques (se hace referencia a la pila de bloques señalándola con el dedo) para construir una torre sobre el tablero de melamina. Aunque los bloques son del juego Jenga, la prueba no se trata de jugar Jenga.
(iii) Puedes poner los bloques en esta posición (mostrar horizontal A[1]); en esta (mostrar horizontal B[2]); o en esta (mostrar vertical). También está permitido quitar los bloques y volverlos a poner en las combinaciones que quieras.
(iv) Si la torre colapsa, puedes reintentarlo cuantas veces quieras, siempre y cuando no haya terminado el tiempo.
(v) Si estás conforme con la altura de tu torre puedes acabar antes del tiempo estipulado. No es obligatorio usar todos los bloques.

II) Single Collapse

(i) El objetivo es construir la torre más alta que puedas en un periodo de 10 minutos.
(ii) Utiliza los bloques (se hace referencia a la pila de bloques señalándola con el dedo) para construir una torre sobre el tablero de melamina. Aunque los bloques son del juego Jenga, la prueba no se trata de jugar Jenga.
(iii) Puedes poner los bloques en esta posición (mostrar horizontal A); en esta (mostrar horizontal B); o en esta (mostrar vertical). También está permitido quitar los bloques y volverlos a poner en las combinaciones que quieras.
(iv) Si la torre colapsa, la prueba termina sin importar el tiempo residual.
(v) Si estás conforme con la altura de tu torre puedes acabar antes del tiempo estipulado. No es obligatorio usar todos los bloques.

III) Record

(i) El objetivo es construir la torre más alta que puedas en un periodo de 10 minutos.
(ii) La marca roja que ves aquí (señalar con el dedo la marca de referencia en el poste de madera) indica el récord de la torre más alta construida hasta ahora por otros estudiantes que han participado en la prueba.
(iii) Utiliza los bloques (se hace referencia a la pila de bloques señalándola con el dedo) para construir una torre sobre el tablero de melamina. Aunque los bloques son del juego Jenga, la prueba no se trata de jugar Jenga.
(iv) Puedes poner los bloques en esta posición (mostrar horizontal A); en esta (mostrar horizontal B); o en esta (mostrar vertical). También está permitido quitar los bloques y volverlos a poner en las combinaciones que quieras.
(v) Si la torre colapsa, puedes reintentarlo cuantas veces quieras, siempre y cuando no haya terminado el tiempo.
(vi) Si estás conforme con la altura de tu torre puedes acabar antes del tiempo estipulado. No es obligatorio usar todos los bloques.

IV) Combined condition: Record-Single Collapse

(i) El objetivo es construir la torre más alta que puedas en un periodo de 10 minutos.
(ii) La marca roja que ves aquí (señalar con el dedo la marca de referencia en el poste de madera) indica el récord de la torre más alta construida hasta ahora por otros estudiantes que han participado en la prueba.
(iii) Utiliza los bloques (se hace referencia a la pila de bloques señalándola con el dedo) para construir una torre sobre el tablero de melamina. Aunque los bloques son del juego Jenga, la prueba no se trata de jugar Jenga.
(iv) Puedes poner los bloques en esta posición (mostrar horizontal A); en esta (mostrar horizontal B); o en esta (mostrar vertical). También está permitido quitar los bloques y volverlos a poner en las combinaciones que quieras.
(v) Si la torre colapsa, la prueba termina sin importar el tiempo residual.
(vi) Si estás conforme con la altura de tu torre puedes acabar antes del tiempo estipulado. No es obligatorio usar todos los bloques.

---

[1] *Horizontal A*: mayor área de contacto, menor altura.

[2] *Horizontal B*: menor área de contacto, mayor altura.

Instructions are written in the order in which they were stated prior to the beginning of the task. All participants were told after instructions were given, that if any questions arose, they were allowed to ask and clarify doubts throughout the building procedure.

S2. Collapse criteria

To consider the undesired loss of height as a collapse three criteria had to be met: i) the loss had to be of at least a quarter of the height constructed, and ii) had to involve a quarter of the pieces that made up the tower, iii) which had to be built with at least 10 blocks.

S3. How the record was determined

Participants from the R and RSC condition were informed that the red mark on a wooden post beside the building board indicated the tower height achieved by a previous participant as the tallest across all previous trials; such information was spurious. The height of the red mark was determined based on a pilot study ($n = 12$) in which participants were presented with one of three "record" references: 90 cm, 120 cm, and 150 cm. Participants presented with the 90 cm record ($n = 4$) reported it was easy to reach, while those presented with a 150 cm record ($n = 4$) reported that they perceived it as unfeasible and preferred to ignore it. Thus, we chose a middle point (120 cm) to provide a record that was not too easy nor too difficult to achieve.

S4. Translation in Spanish of the Sensation Seeking Scale: Escala de Búsqueda de Sensaciones

Instrucciones: cada uno de los elementos que vienen abajo tiene dos opciones, A y B. Por favor indica (encierra en un círculo) la respuesta que mejor describe tus preferencias o sentimientos.

En algunos casos, puede ser que ambos elementos te parezcan apropiados. Elige por favor el que mejor lo haga. En otros casos, puede ser que no te guste ninguna de las opciones, si es así, marca el elemento que te disguste menos. Intenta responder todos y cada uno de los elementos.

Es importante responder a todos los elementos con solo una elección, ya sea A o B. Estamos interesados en tus preferencias o inclinaciones, no en tu opinión sobre estas cuestiones ni en la opinión que se supone debes de tener. No existen respuestas correctas o incorrectas. Se franco y danos un juicio honesto sobre ti.

	A	B
1.	Me gustan las fiestas "alocadas" y desinhibidas	Prefiero las fiestas tranquilas con buena conversación
2.	Hay películas que disfruto ver por segunda o tercera vez	No tolero ver películas que ya he visto antes
3.	Frecuentemente sueño con ser un montañista	No entiendo a la gente que arriesga la vida escalando montañas
4.	Me disgustan los olores corporales	Me gustan algunos olores corporales que son como de tierra
5.	Me aburre frecuentar a las mismas personas	Me gusta la sensación de comodidad de los buenos amigos
6.	Me gusta explorar una ciudad desconocida o una parte nueva de la ciudad por mi cuenta, incluso si esto significa perderme	Prefiero tener un guía en un lugar que no conozco
7.	Me disgusta la gente que hace o dice cosas sólo para escandalizar o molestar a otros	Cuando puedes predecir todo lo que una persona dice o hace, ésta se vuelve aburrida
8.	Usualmente no disfruto una película u obra si puedo predecir lo que pasará	No me molesta ver una película u obra donde puedo predecir lo que va a pasar
9	He probado la mariguana o me gustaría probarla	Nunca fumaría mariguana
10.	No me gustaría probar una droga que me cause efectos extraños o peligrosos	Me gustaría probar alguna de las nuevas drogas que te hacen alucinar
11.	Una persona sensata evita actividades que son peligrosas	A veces hago cosas que me dan un poco de miedo
12.	No me gustan los "swingers" (gente desinhibida que acostumbra intercambiar parejas sexuales)	Disfruto la compañía de "swingers" (gente desinhibida que acostumbra intercambiar parejas sexuales)
13.	Los estimulantes me ponen incómodo	Con frecuencia me gusta sentir el efecto de algunas drogas (alcohol o mariguana)

14.	Me gusta probar comidas nuevas que no había probado antes	Me gusta mantener la cocina ordenada y así evitar desilusiones y molestias
15.	Disfruto ver películas caseras o que otros me cuenten de sus viajes	Ver películas caseras u oír de algún viaje de otra persona me aburre enormemente
16.	Me gustaría practicar el deporte de esquí acuático	No me gustaría aprender esquí acuático
17.	Me gustaría intentar surfear	No me gustaría intentar surfear
18.	Me gustaría salir de viaje sin planes, rutas definitivas o un programa de actividades	Cuando salgo de viaje planeo mi ruta e itinerario con bastante cuidado
19.	Prefiero hacerme amigo de gente “centrada”	Me gustaría tener amigos en grupos más marginales como artistas o “punks”
20.	No me gustaría aprender a pilotear un avión	Me gustaría aprender a pilotear un avión
21.	Prefiero la superficie del agua que las profundidades	Me gustaría practicar buceo
22.	Me gustaría conocer personas homosexuales	Prefiero evitar la compañía de alguien que sospecho es homosexual
23.	Me gustaría intentar saltar en paracaídas	Nunca intentaría saltar de un avión, con o sin paracaídas
24.	Prefiero amigos que son emocionantemente impredecibles	Me gustan los amigos que son predecibles y de fiar
25.	No me interesa una experiencia sólo por el simple hecho de tenerla	Me gusta tener experiencias y sensaciones nuevas y emocionantes, incluso si dan un poco miedo, son poco convencionales o ilegales
26.	La esencia del buen arte está en su claridad, simetría de formas y armonía de colores	Con frecuencia encuentro belleza en los colores “chocantes” y las formas irregulares de las pinturas modernas
27.	Disfruto pasar el tiempo en el entorno familiar de casa	Me pongo inquieto si debo quedarme en casa por algún periodo de tiempo
28.	Me gusta echarme clavados desde el trampolín más alto	No me gusta lo que siento al pararme en el trampolín más alto (o ni me acerco)
29.	Me gusta salir con miembros del sexo opuesto que son físicamente atractivos	Me gusta salir con miembros del sexo opuesto que comparten mis valores
30.	Un exceso de alcohol arruina una fiesta porque la gente se pone ruidosa y bulliciosa	La abundancia de alcohol es la clave para una buena fiesta
31.	El pecado social más grave es ser descortés	El pecado social mas grave es ser aburrido
32.	Una persona debería de tener bastante experiencia sexual antes del matrimonio	Es mejor si un matrimonio comienza su experiencia sexual al casarse

33.	Aun si tuviera dinero, no me gustaría relacionarme con gente rica que presume su dinero	Podría imaginarme buscando placeres en el mundo junto con gente rica que presume su dinero
34.	Me gustan las personas astutas y ocurrentes aun cuando a veces insulten a otros	No me gusta la gente que se divierte a costa de los sentimientos de otros
35.	Hay demasiadas escenas de sexo en las películas	Disfruto las escenas de sexo en las películas
36.	Me siento mejor después de tomar un par de tragos	Algo está mal en la gente que necesita del alcohol para sentirse bien
37.	La gente se debe de vestir según algún estándar de buen gusto, pulcritud y estilo	La gente debería de vestir a su manera aun cuando la combinación de prendas resulte extraña
38.	Navegar largas distancias en un bote pequeño es temerario	Me gustaría navegar largas distancias en un barco pequeño pero resistente
39.	No tengo paciencia con las personas sosas y aburridas	Encuentro algo interesante en prácticamente cada persona con la que hablo
40.	Bajar una pendiente montañosa en esquíes es una buena forma de acabar en muletas	Pienso que disfrutaría la sensación de bajar una montaña esquiando rápidamente

S5. Table of additional descriptors of behavior in the TBT

Mean ± standard deviation or frequency (% percentage) of the descriptors of behavior for each of the TBT conditions.

Descriptor of behavior	Baseline (n = 29)	Single Collapse (n = 30)	Record (n = 28)	Record-Single Collapse (n = 30)
Maximum height (cm)	85.43 ± 28.24	81.2 ± 37.71	89.66 ± 25.61	85.82 ± 27.13
Addition rate (s per block added)	5.08 ± 2.6	4.76 ± 1.81	4.54 ± 1.23	5.1 ± 1.63
Duration of the trial (s)	522.73 ± 123.47	414.78 ± 144.62	569.47 ± 73.71	394.15 ± 144.27
Proportion of vertical pieces	0.16 ± 0.26	0.18 ± 0.26	0.23 ± 0.24	0.24 ± 0.28
Number of collapses	0.9 ± 1.08	0.3 ± 0.47	1.54 ± 1.37	0.53 ± 0.51
Early conclusion	13 (44.83%)	16 (53.33%)	8 (28.57%)	10 (33.33%)
Collapse	13 (44.83%)	9 (30.00%)	20 (71.43%)	16 (53.33%)

Note. Maximum Height = tallest tower achieved throughout the trial; Duration of the trial = amount of time spent on the task; Early conclusion = number of participants who chose to finish the trial before the established 10 minutes; Collapse = number of participants who had at least one collapse.

S6. Non-significant associations between fixed height gain and the two other risk-related measures

Scatter plots for the i) Baseline, ii) Record, and iii) Record-Single Collapse conditions of the TBT in relation to the BART and the SSS measures. Filled circles show participants' fixed height gain on the x-axis and the adjusted average number of pumps or the sensation seeking score on the y-axis. Blue lines show the linear association between the two variables of each plot and the gray area surrounding such lines indicates the confidence intervals. The fixed height gain is an index that equates to the height gained per block added adjusted for the duration of the trial.

i) Baseline condition

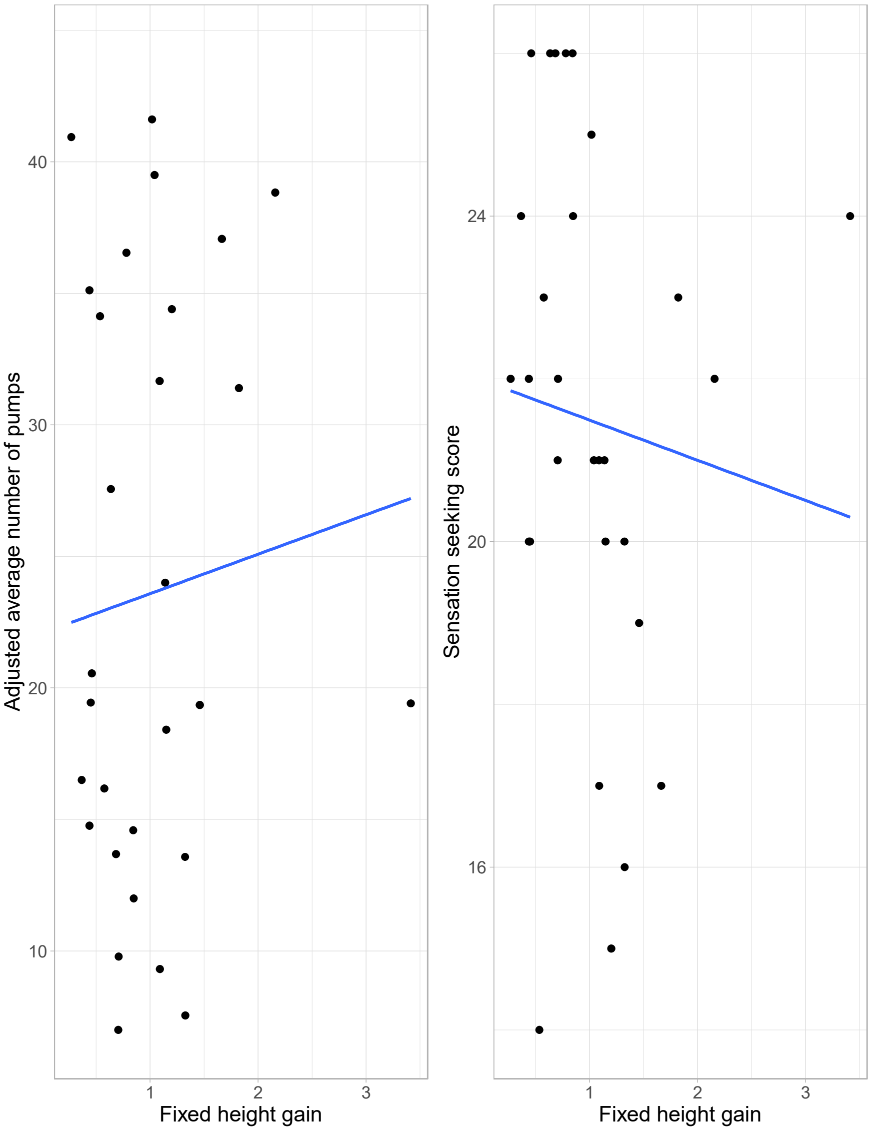

ii) Record condition

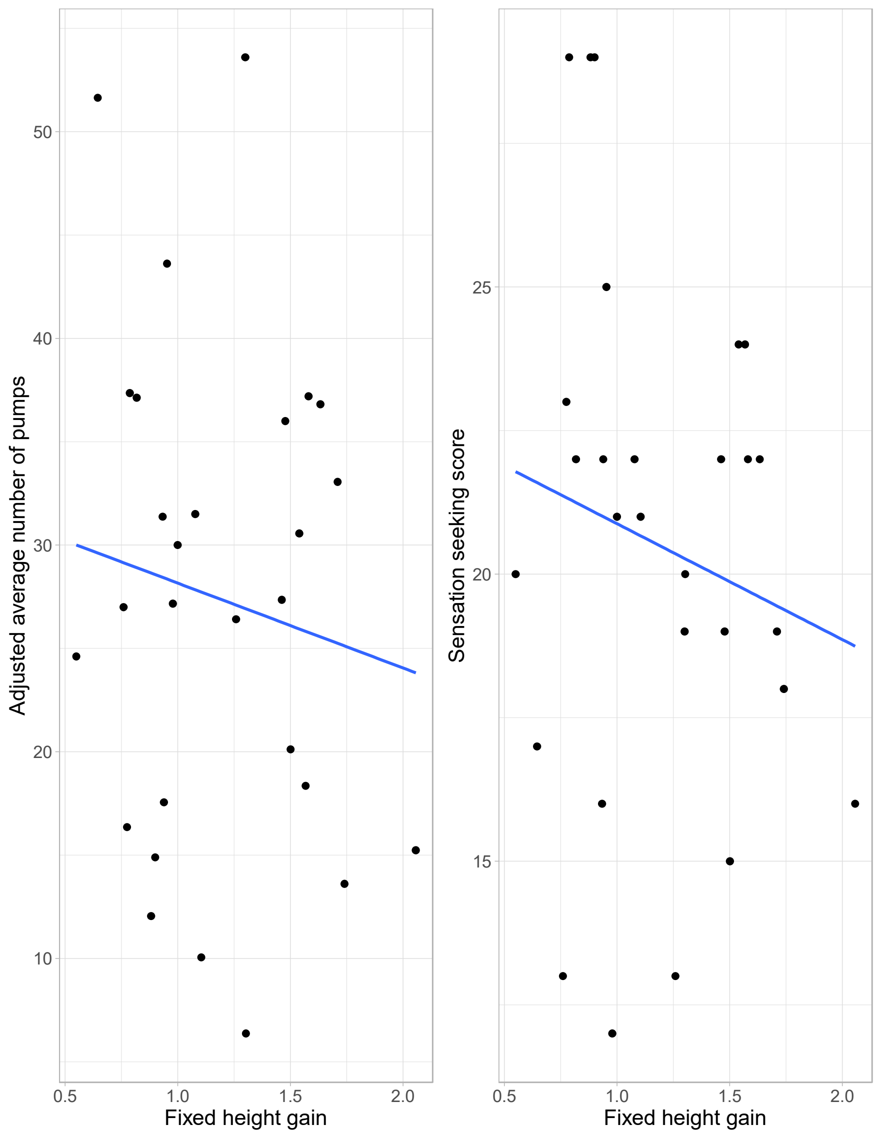

iii) Record-Single Collapse condition

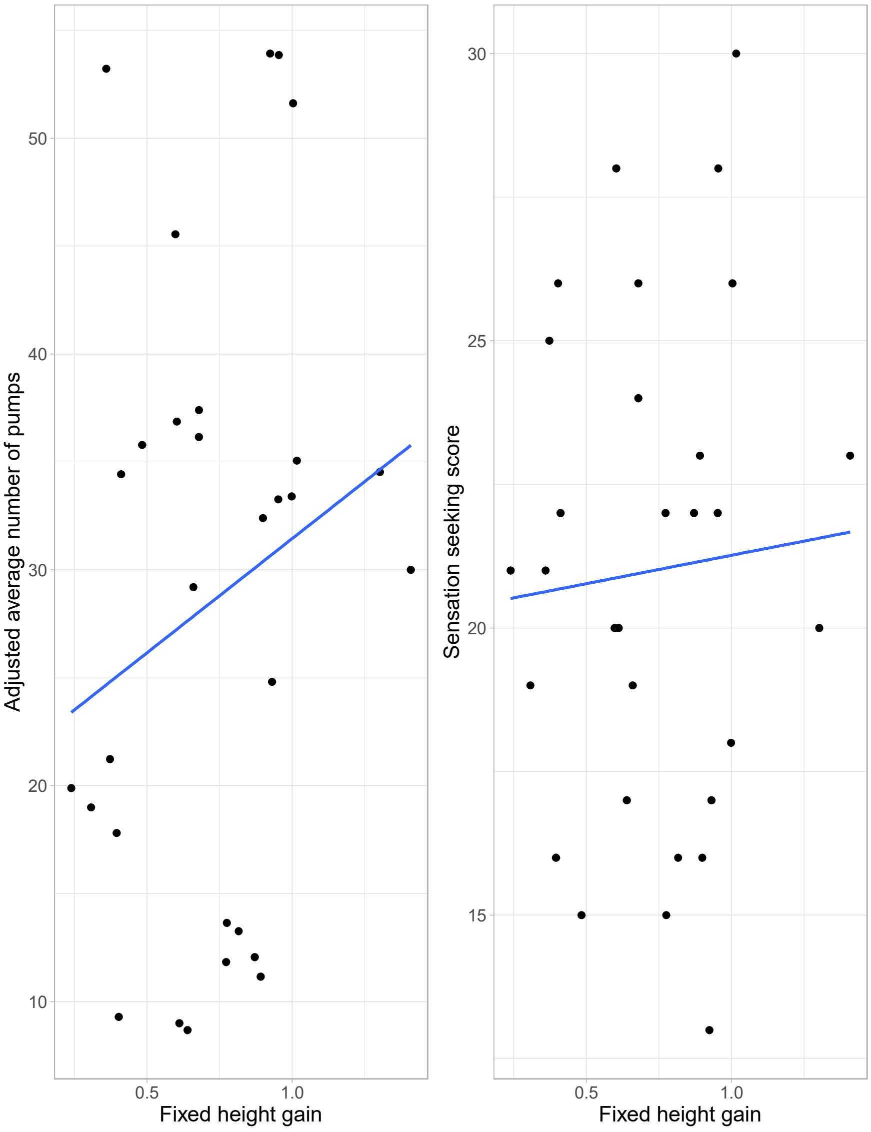

Supplement: Supplementary file 1 [file behavsci-12-00325-s001.zip › behavsci-1817294-supplementary.pdf]
